# Successful Bailout of Guidewire Entrapment Within a Coronary Stent Using a Tornus Penetration Catheter: A Case Report

**DOI:** 10.7759/cureus.116956

**Published:** 2026-09-26

**Authors:** Tomohide Sato, Eiji Horio, Kazuhisa Kodama, Tomohiro Sakamoto, Junjiroh Koyama

**Affiliations:** 1 Cardiology, Saiseikai Kumamoto Hospital Cardiovascular Center, Kumamoto, JPN

**Keywords:** bailout technique, coronary stent, guidewire entrapment, percutaneous coronary intervention, tornus catheter

## Abstract

Guidewire entrapment within an implanted coronary stent is a rare but potentially serious complication of percutaneous coronary intervention (PCI), and its management can be technically challenging. An 81-year-old woman presenting with acute myocardial infarction underwent PCI for a severely stenotic lesion in the right coronary artery. After drug-eluting stent implantation and post-dilatation, intravascular ultrasound (IVUS) revealed deformation at the distal edge of the stent. During withdrawal of the IVUS catheter, the catheter and guidewire became entangled with the deformed stent. Although the IVUS catheter was successfully removed with careful manipulation, the guidewire remained firmly entrapped within the stent and could not be retrieved despite repeated attempts. A Tornus penetration catheter was subsequently advanced over the entrapped guidewire using rotational manipulation toward the site of entrapment. When the catheter reached the entrapped segment, the guidewire was successfully disengaged from the stent and retrieved. Following successful bailout, distal stent fracture and proximal coronary dissection were identified, requiring additional stent implantation. This case demonstrates a potential bailout technique for guidewire entrapment within a coronary stent. Rotational advancement of a penetration catheter to the site of entrapment may facilitate disengagement of an entrapped guidewire when standard manipulation is unsuccessful.

## Introduction

Guidewire entrapment is a rare but potentially serious complication of percutaneous coronary intervention (PCI) [1,2]. An entrapped guidewire may result in wire fracture, coronary artery injury, thrombosis, myocardial ischemia, or the need for surgical removal [1,2]. Therefore, safe and effective percutaneous retrieval should be attempted whenever feasible.

Several techniques for retrieving entrapped guidewires have been described, including the use of balloon catheters, snares, multiple guidewires, guide-extension catheters, and microcatheters [1,2]. Rotational microcatheters, including the Tornus catheter, may facilitate guidewire release by advancing toward the site of entrapment [1,3]. However, guidewire entrapment within a deployed and deformed coronary stent can be particularly challenging because excessive traction may further deform the stent or result in guidewire fracture.

Although the use of rotational microcatheters for guidewire entrapment has been described, experience with their use for guidewire entrapment within a deployed and deformed coronary stent remains limited. Here, we report a case of guidewire entrapment within a deformed coronary stent during PCI in which the entrapped guidewire was successfully released by rotationally advancing a Tornus penetration catheter to the site of entrapment. The aims of this report are to describe this rotational bailout technique, discuss the force dynamics that may have contributed to guidewire disengagement, and highlight important safety considerations, including secondary procedural complications associated with device manipulation.

## Case presentation

An 81-year-old woman with a history of hypertension developed sudden-onset chest pain while at a golf course and was transported to our hospital by ambulance. The chest pain persisted on arrival, and she had no previous history of similar symptoms. Her regular medications were amlodipine besylate 2.5 mg and esomeprazole magnesium hydrate 20 mg.

On admission, she was alert, with a blood pressure of 151/72 mmHg, heart rate of 51 beats/min, respiratory rate of 24 breaths/min, body temperature of 36.0°C, and oxygen saturation of 100% while receiving oxygen at 1 L/min. Physical examination revealed no remarkable findings. The principal laboratory findings are summarized in Table 1.

**Table 1:** Laboratory findings on admission.

Tests performed	Result	Reference range
White blood cell count (WBC), /μL	12,200	3,300-8,600
Red blood cell count (RBC), ×10^4^/μL	405	386-492
Hemoglobin (Hb), g/dL	12.8	11.6-14.8
Hematocrit (Ht), %	38.2	35.1-44.4
Platelet count (Plt), ×10^4^/μL	22.7	15.8-34.8
Albumin (Alb), g/dL	3.8	4.1-5.1
Blood urea nitrogen (BUN), mg/dL	14.7	8-20
Creatinine (Cr), mg/dL	0.60	0.65-1.07
Sodium (Na), mEq/L	141	138-145
Potassium (K), mEq/L	4.07	3.6-4.8
Chloride (Cl), mEq/L	105	101-108
Total bilirubin (T-Bil), mg/dL	0.5	0.4-1.5
Aspartate aminotransferase (AST), U/L	22	13-30
Alanine aminotransferase (ALT), U/L	10	7-23
Lactate dehydrogenase (LDH), U/L	204	124-222
Creatine kinase (CK), U/L	167	41-153
Creatine kinase-MB (CK-MB), ng/mL	11	≤5
Troponin I (TnI), pg/mL	169	≤26.2
B-type natriuretic peptide (BNP), pg/mL	22.9	≤18.4
Total cholesterol (TC), mg/dL	195	142-248
High-density lipoprotein cholesterol (HDL-C), mg/dL	72	38-90
Triglycerides (TG), mg/dL	98	40-234
Low-density lipoprotein cholesterol (LDL-C), mg/dL	104	65-163
Blood glucose, mg/dL	119	73-109
Hemoglobin A1c (HbA1c), %	6.1	4.9-6.0
Prothrombin time (PT), %	115.2	80-120
Activated partial thromboplastin time (APTT), sec	24.8	24-36

A 12-lead electrocardiogram showed ST-segment elevation in leads II, III, and aVF, with reciprocal ST-segment depression in lead aVL (Figure 1).

**Figure 1:**
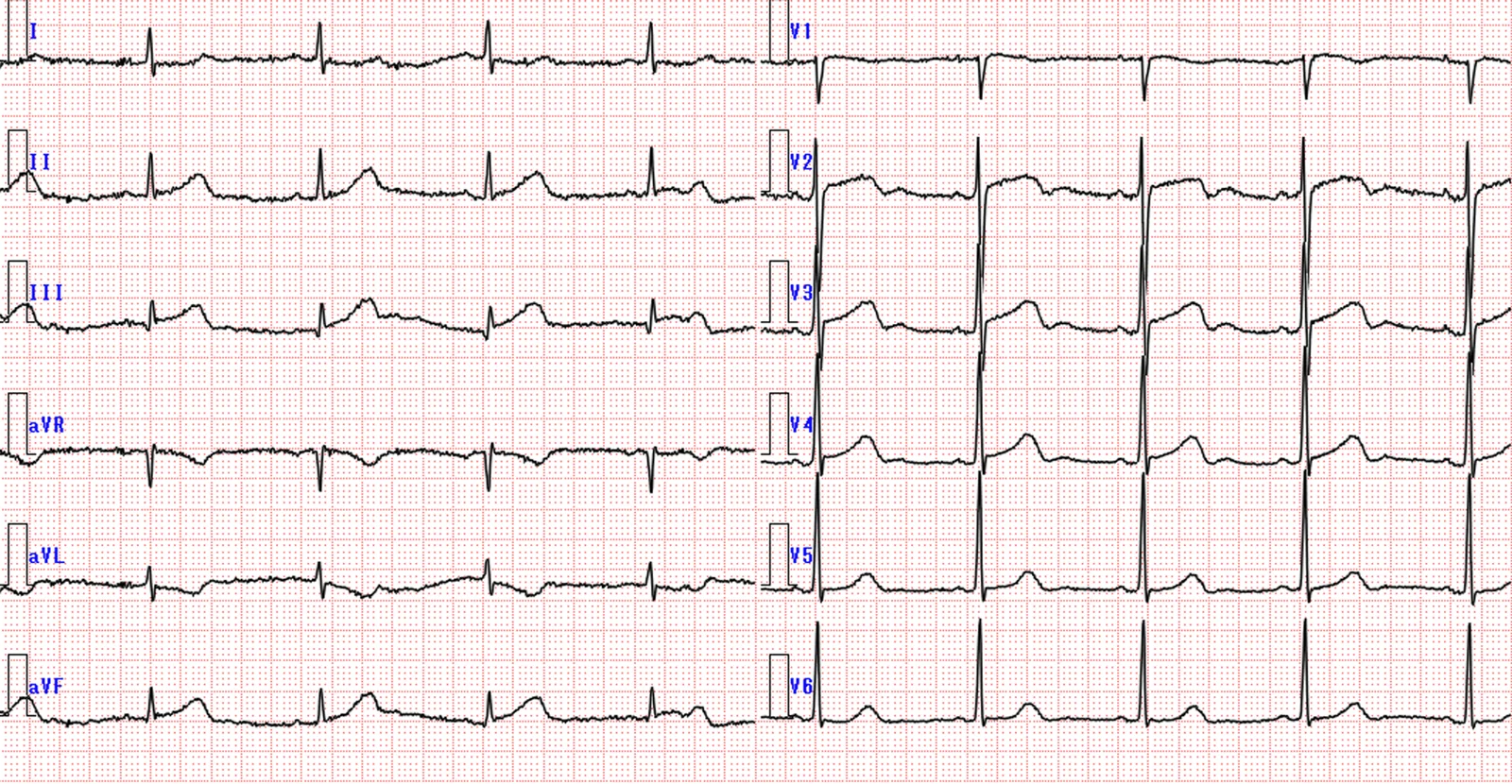
Twelve-lead electrocardiogram on admission. The electrocardiogram showed sinus rhythm with ST-segment elevation in leads II, III, and aVF, accompanied by reciprocal ST-segment depression in lead aVL.

Chest radiography showed mild cardiomegaly without significant pulmonary congestion (Figure 2).

**Figure 2:**
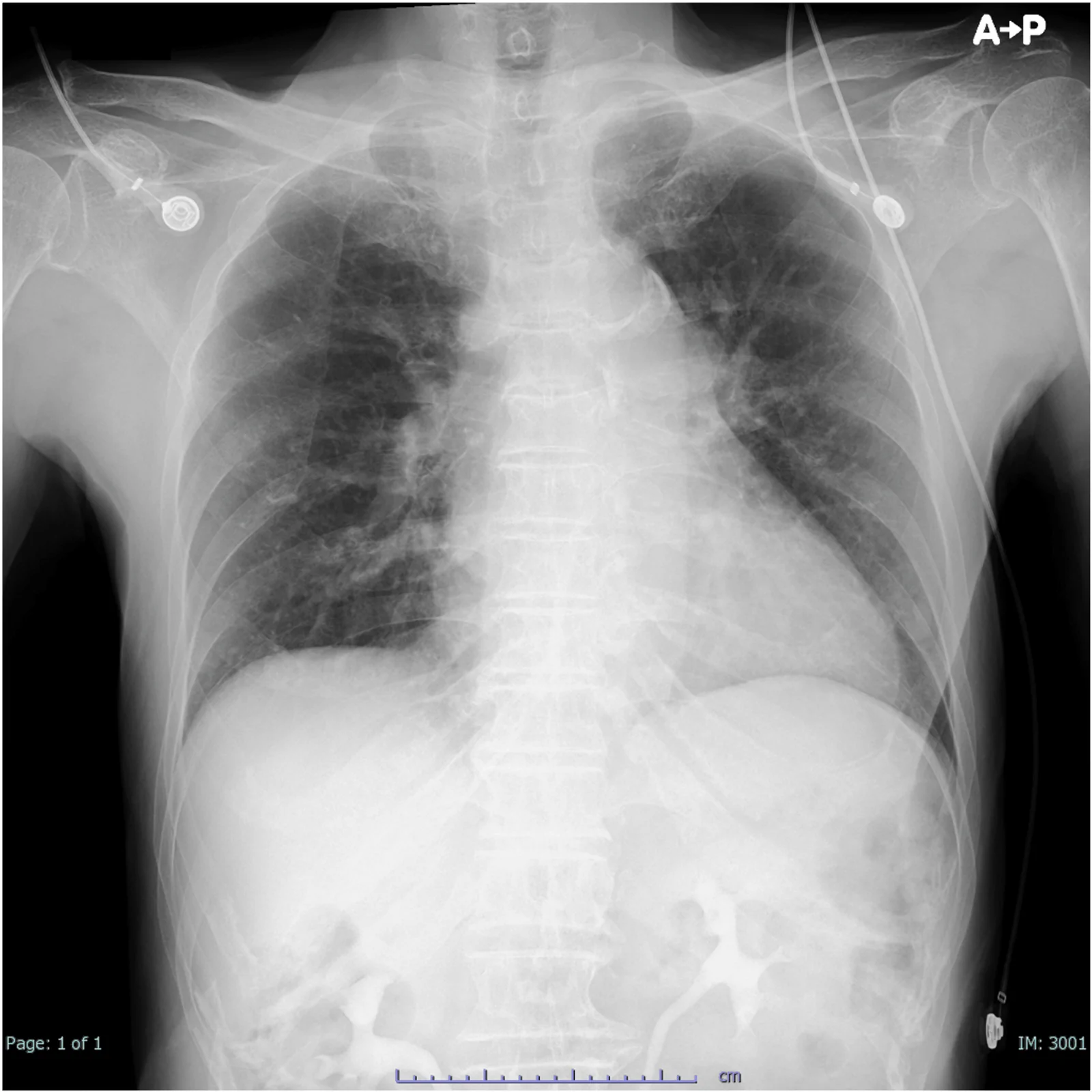
Chest radiograph on admission. Chest radiography showed mild cardiomegaly without significant pulmonary congestion.

Transthoracic echocardiography showed a left ventricular ejection fraction of 57% with inferior wall hypokinesis (Figure 3).

**Figure 3:**
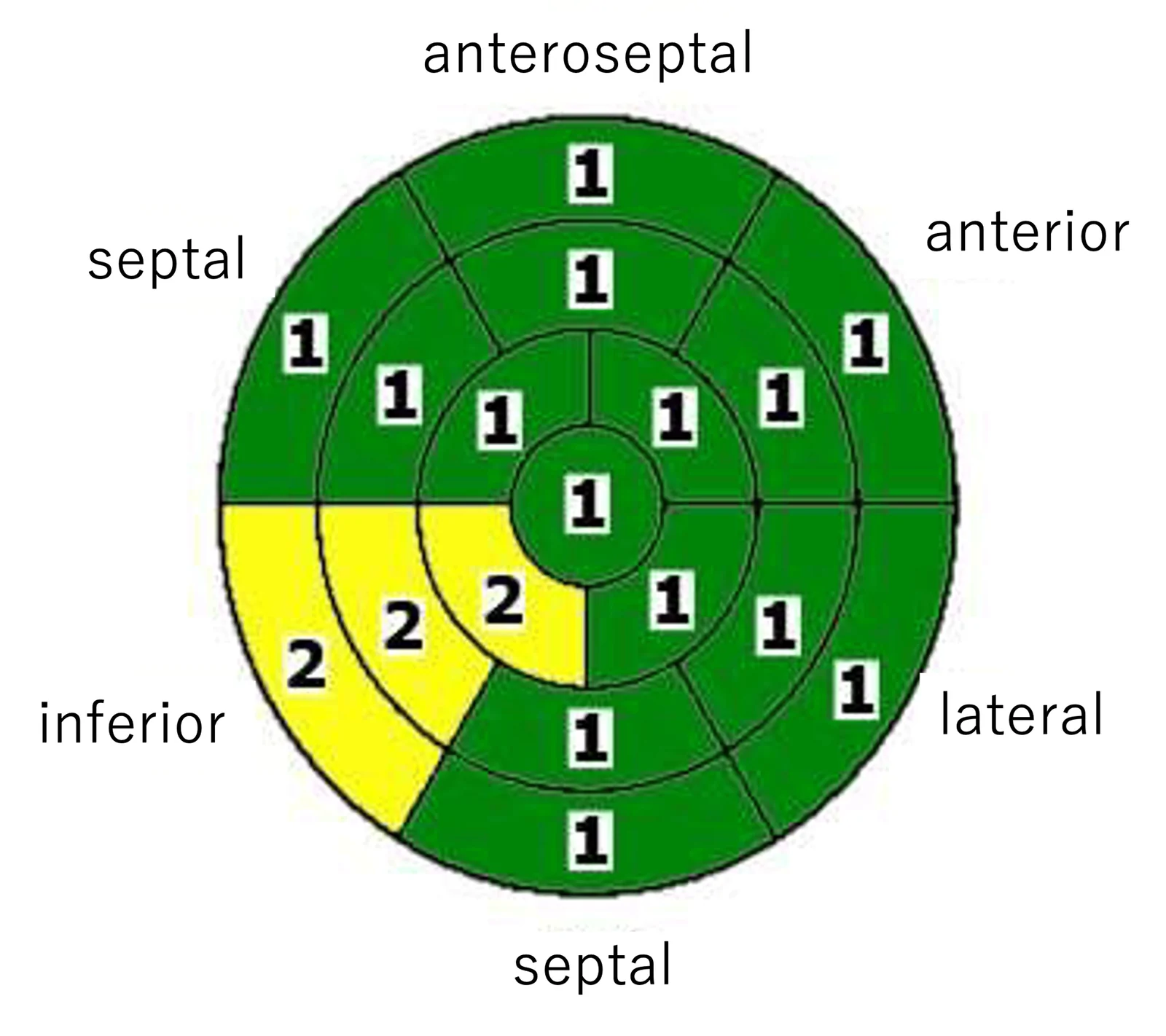
Schematic representation of left ventricular wall motion on admission. The bull's-eye diagram shows hypokinesis of the inferior wall (yellow). Wall-motion scores were defined as follows: 1, normal; and 2, hypokinesis. The figure was created by the authors using Microsoft PowerPoint (Microsoft Corp., Redmond, WA, USA) based on the findings of transthoracic echocardiography.

Based on these findings, acute myocardial infarction was diagnosed, and emergency cardiac catheterization was performed. Before PCI, loading doses of aspirin 200 mg and prasugrel 20 mg were administered. Unfractionated heparin (5,000 U) was initially administered intravenously, with additional doses given as needed based on serial activated clotting time measurements. No glycoprotein IIb/IIIa inhibitor was used.

Emergency coronary angiography revealed 90% stenosis in segment 2 of the right coronary artery (Figure 4), and PCI was subsequently performed.

**Figure 4:**
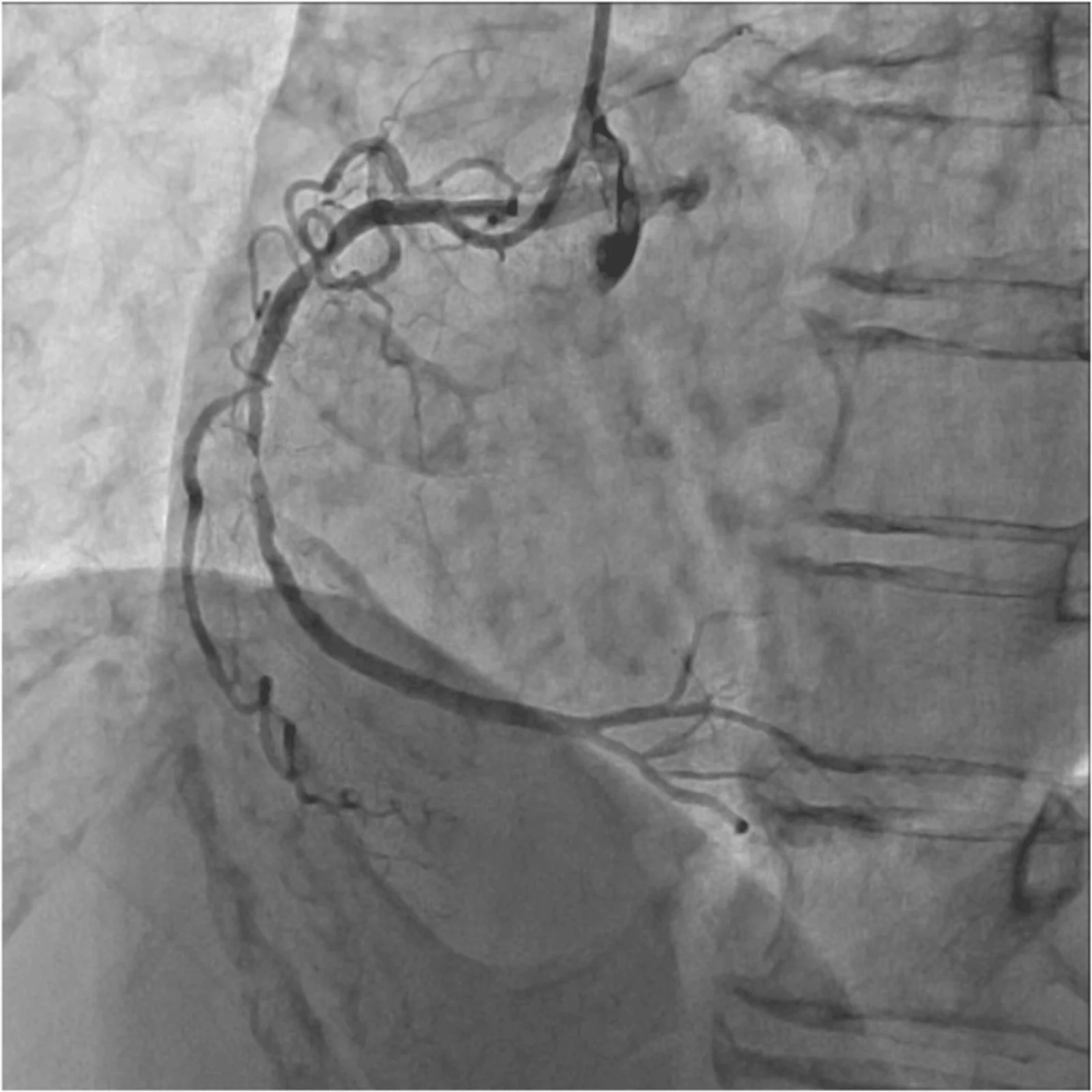
Initial coronary angiography. Emergency coronary angiography revealed 90% stenosis in segment 2 of the right coronary artery.

A JR 4.0 guiding catheter was engaged in the right coronary artery, and a 0.014-inch ASAHI SION blue coronary guidewire (ASAHI INTECC CO., LTD., Seto, Aichi, Japan), with a tip load of 0.5 gf and silicone and hydrophilic coatings, was advanced across the target lesion. After lesion assessment with an AltaView intravascular ultrasound (IVUS) catheter (Terumo Corporation, Tokyo, Japan), predilatation was performed using a 2.5 × 15-mm scoring balloon, followed by implantation of a 2.5 × 38-mm XIENCE Skypoint everolimus-eluting stent (Abbott, Chicago, IL, USA) (Figure 5).

**Figure 5:**
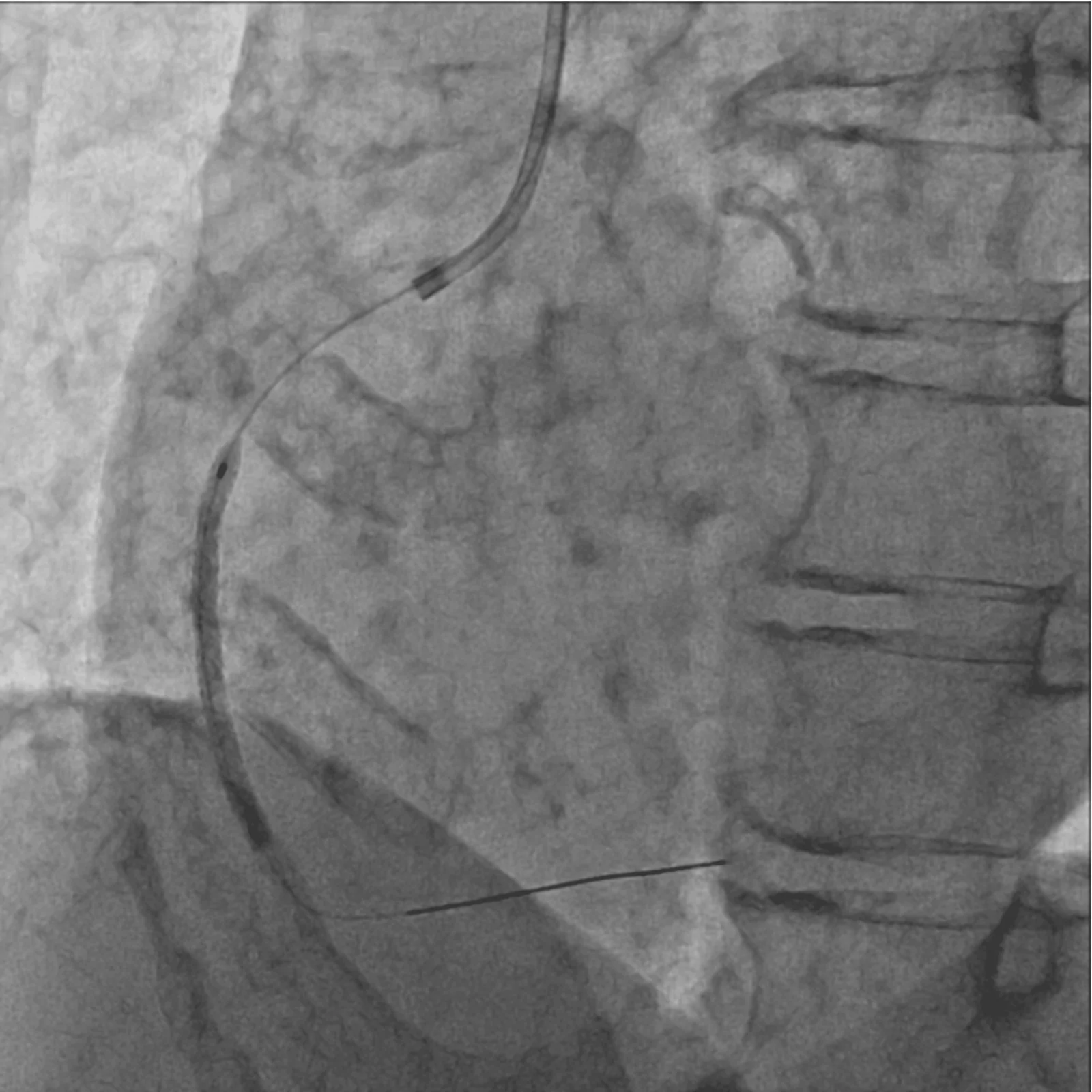
Drug-eluting stent implantation. A 2.5 × 38-mm drug-eluting stent was implanted in the target lesion of the right coronary artery.

Subsequent IVUS examination revealed an area of incomplete stent apposition. Therefore, post-dilatation was performed using a 2.75 × 10-mm NC KAMUI non-compliant balloon (ASAHI INTECC CO., LTD.) at up to 20 atm (Figure 6).

**Figure 6:**
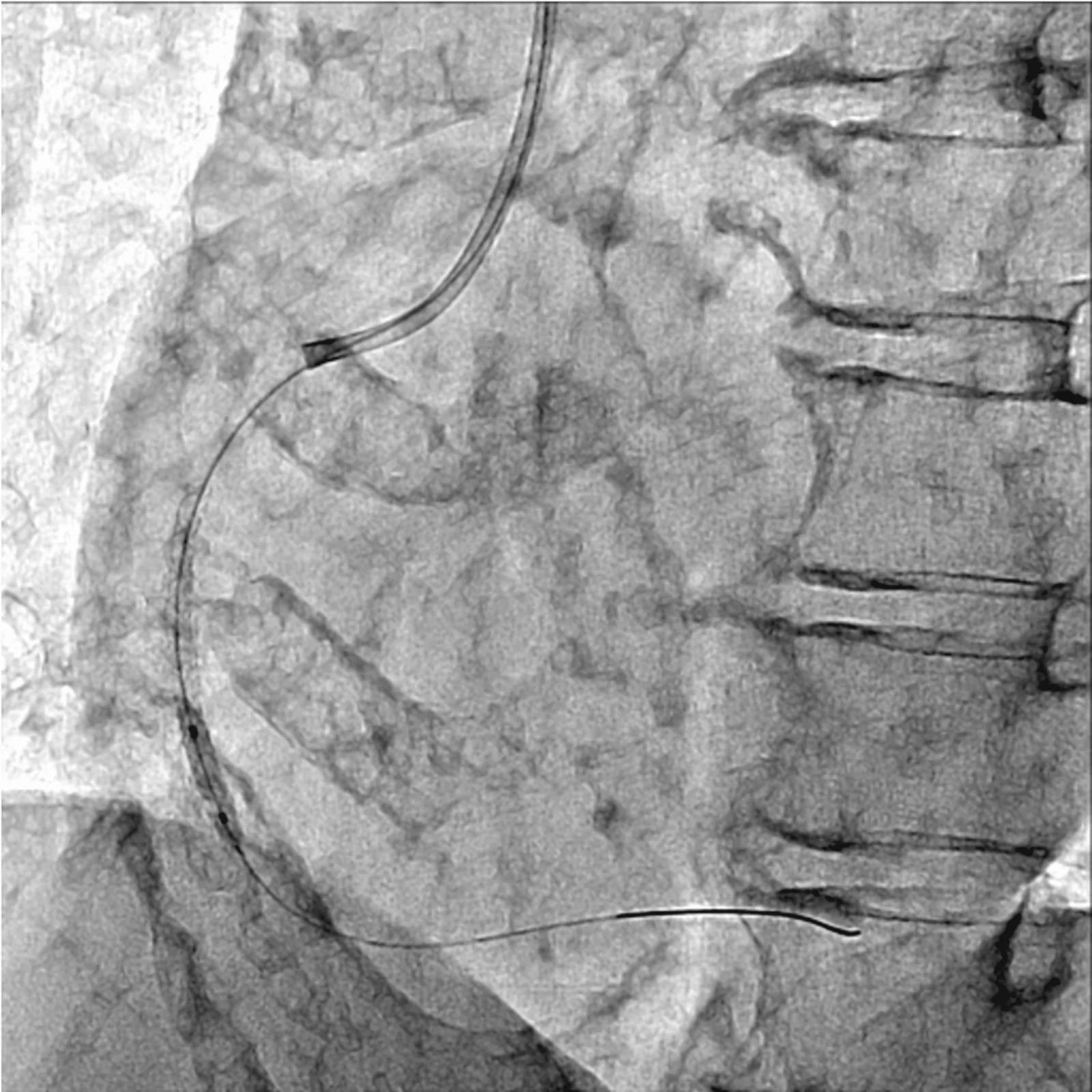
Post-dilatation of the distal portion of the implanted stent. Post-dilatation of the distal portion of the implanted stent was performed using a 2.75 × 10-mm non-compliant balloon.

Further IVUS examination revealed deformation of the stent at its distal edge (Figure 7), and additional stent implantation was considered.

**Figure 7:**
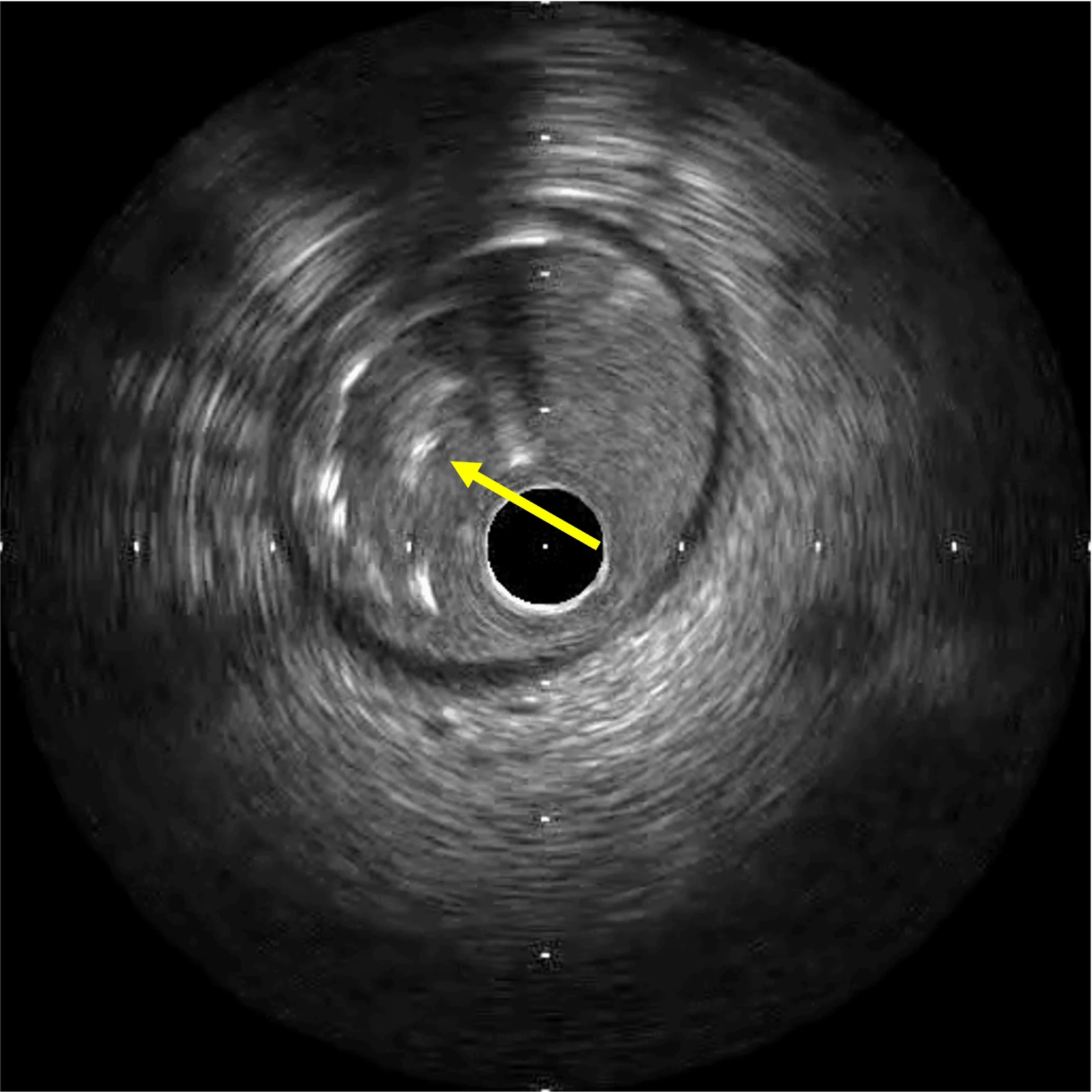
Intravascular ultrasound image showing distal stent deformation. Intravascular ultrasound examination after post-dilatation revealed deformation of the distal portion of the implanted stent (yellow arrow).

Thereafter, marked resistance was encountered during withdrawal of the IVUS catheter. Fluoroscopy demonstrated fixation of the guidewire and further deformation of the distal stent, raising suspicion that the IVUS catheter and guidewire were entangled with the deformed stent. The IVUS catheter was eventually removed by repeated careful manipulation; however, the guidewire remained immobile and could not be retrieved despite traction, confirming persistent guidewire entrapment within the stent (Figure 8).

**Figure 8:**
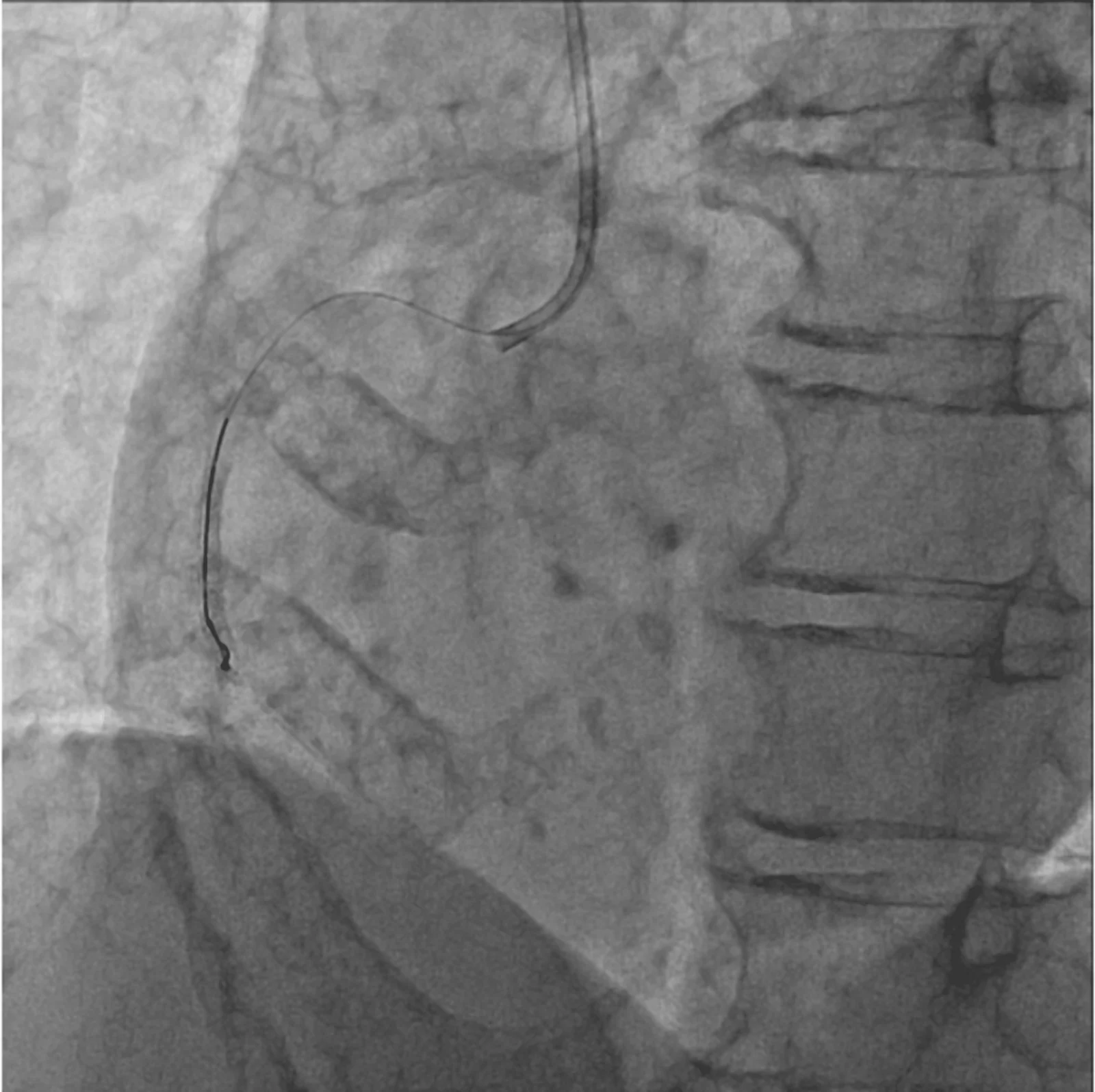
Guidewire entrapment within the coronary stent. The coronary guidewire remained firmly entrapped within the deformed stent after removal of the IVUS catheter. IVUS: intravascular ultrasound

Several attempts were made to release the entrapped guidewire. First, a Zizai microcatheter (Terumo Clinical Supply Co., Ltd., Kakamigahara, Gifu, Japan) was advanced to engage and retrieve the guidewire; however, the guidewire could not be accommodated within the microcatheter (Figure 9).

**Figure 9:**
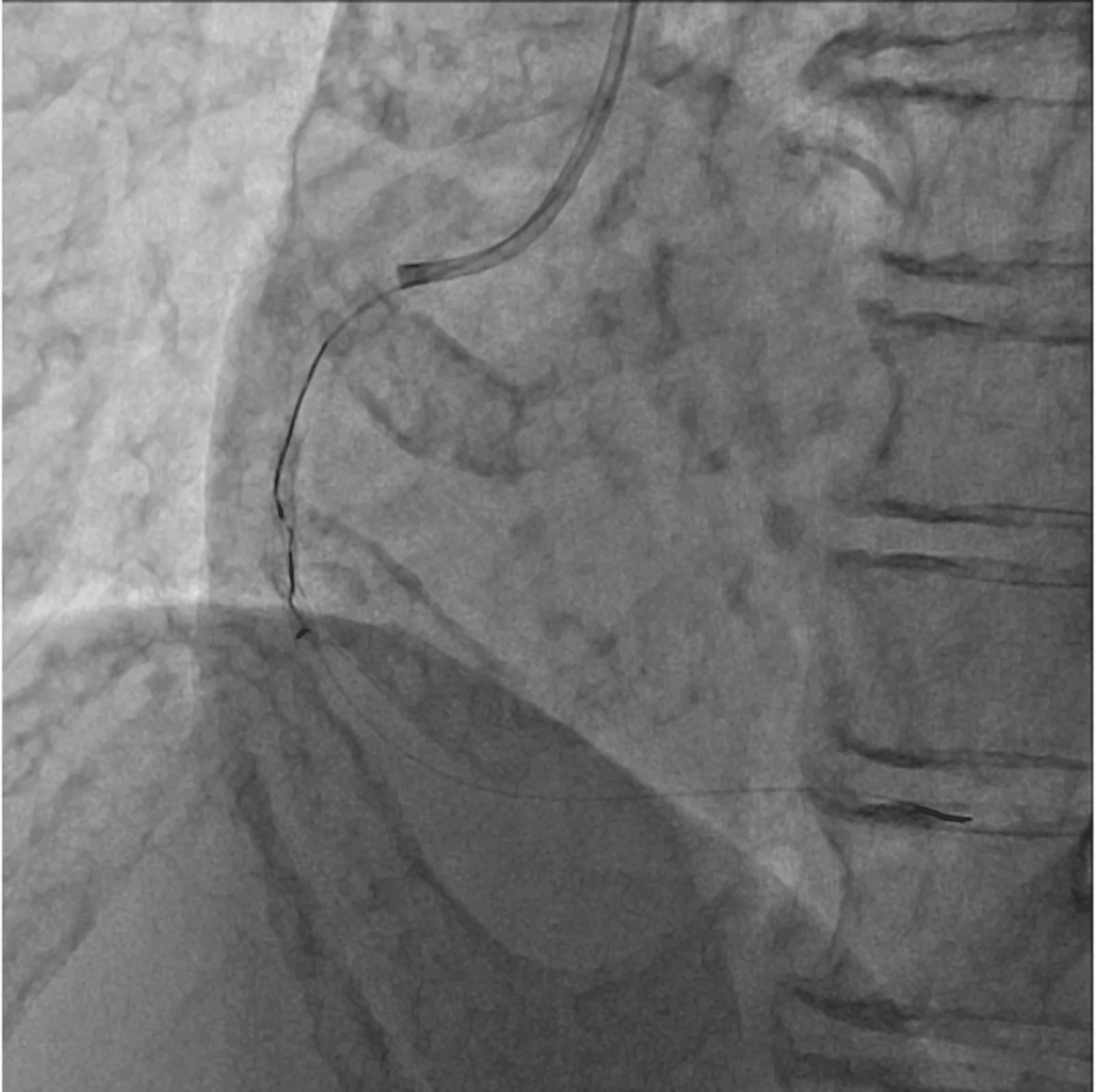
Attempted guidewire retrieval using a microcatheter. A microcatheter was advanced over the entrapped guidewire in an attempt to retrieve the guidewire; however, the guidewire could not be accommodated within the microcatheter.

Next, a ZINRAI 0.75 × 4-mm balloon catheter (Kaneka Medix Corporation, Osaka, Japan) was advanced to the site of entrapment and inflated to its nominal pressure to create sufficient space between the guidewire and the stent (Figure 10).

**Figure 10:**
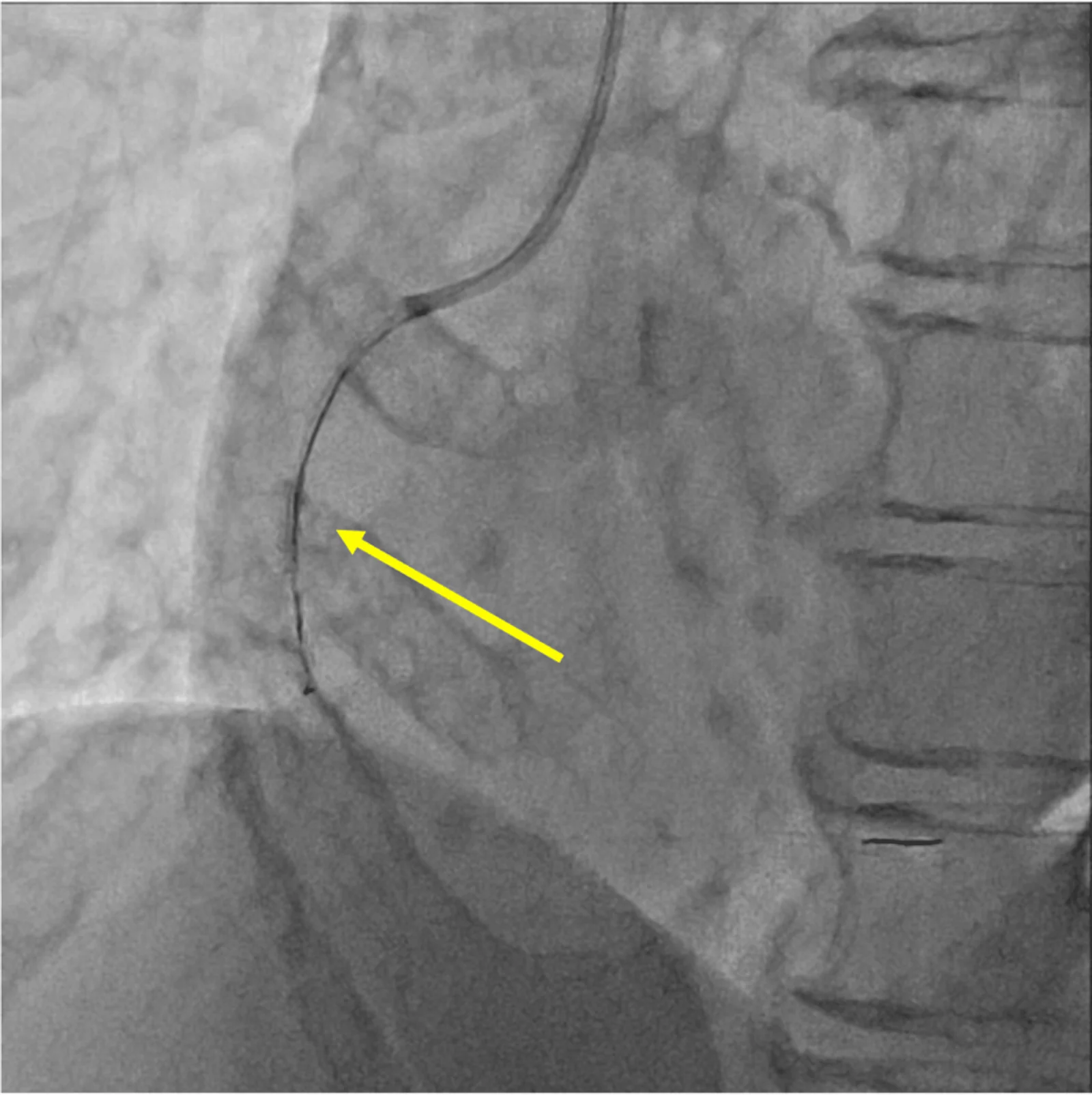
Attempted guidewire release using a small balloon catheter. A 0.75 × 4-mm balloon catheter was advanced to the site of guidewire entrapment and inflated to create space between the guidewire and the stent (yellow arrow). However, the guidewire remained entrapped.

However, the guidewire remained entrapped.

A Tornus penetration catheter (ASAHI INTECC CO., LTD.) was then advanced over the entrapped guidewire. The catheter was progressively advanced using rotational manipulation toward the site where the guidewire was entrapped within the stent. When the Tornus catheter reached the site of entrapment, the guidewire was successfully disengaged from the stent and retrieved (Figure 11).

**Figure 11:**
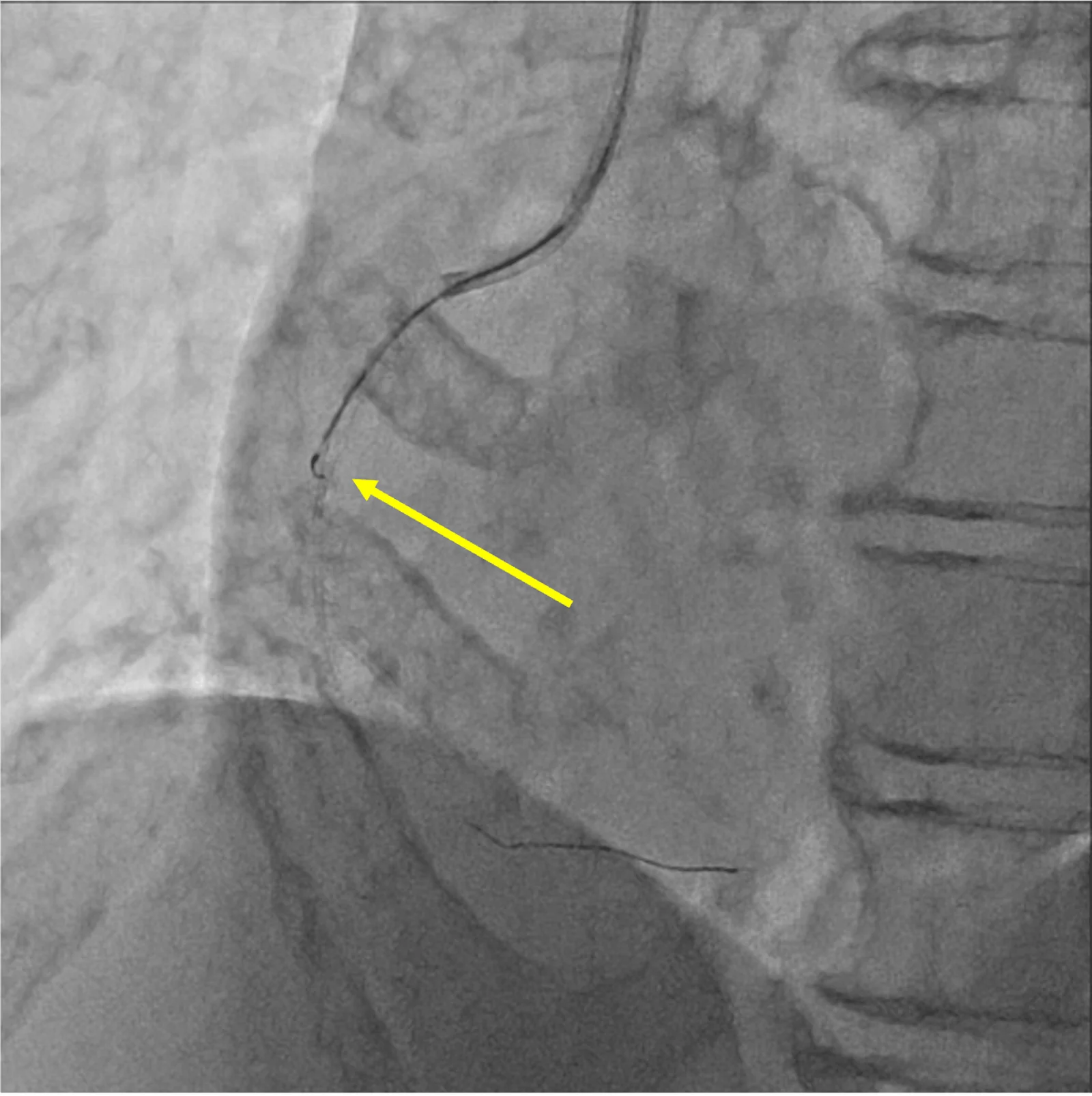
Successful release of the entrapped guidewire using a Tornus penetration catheter. A Tornus penetration catheter was advanced with rotational manipulation over the entrapped guidewire toward the site of entrapment (yellow arrow). When the Tornus catheter reached the entrapment site, the guidewire was successfully disengaged from the stent and retrieved.

After successful bailout, fracture of the distal portion of the previously implanted stent was identified by both fluoroscopy and IVUS; therefore, a 2.75 × 18-mm drug-eluting stent (DES) was implanted at the distal site (Figure 12).

**Figure 12:**
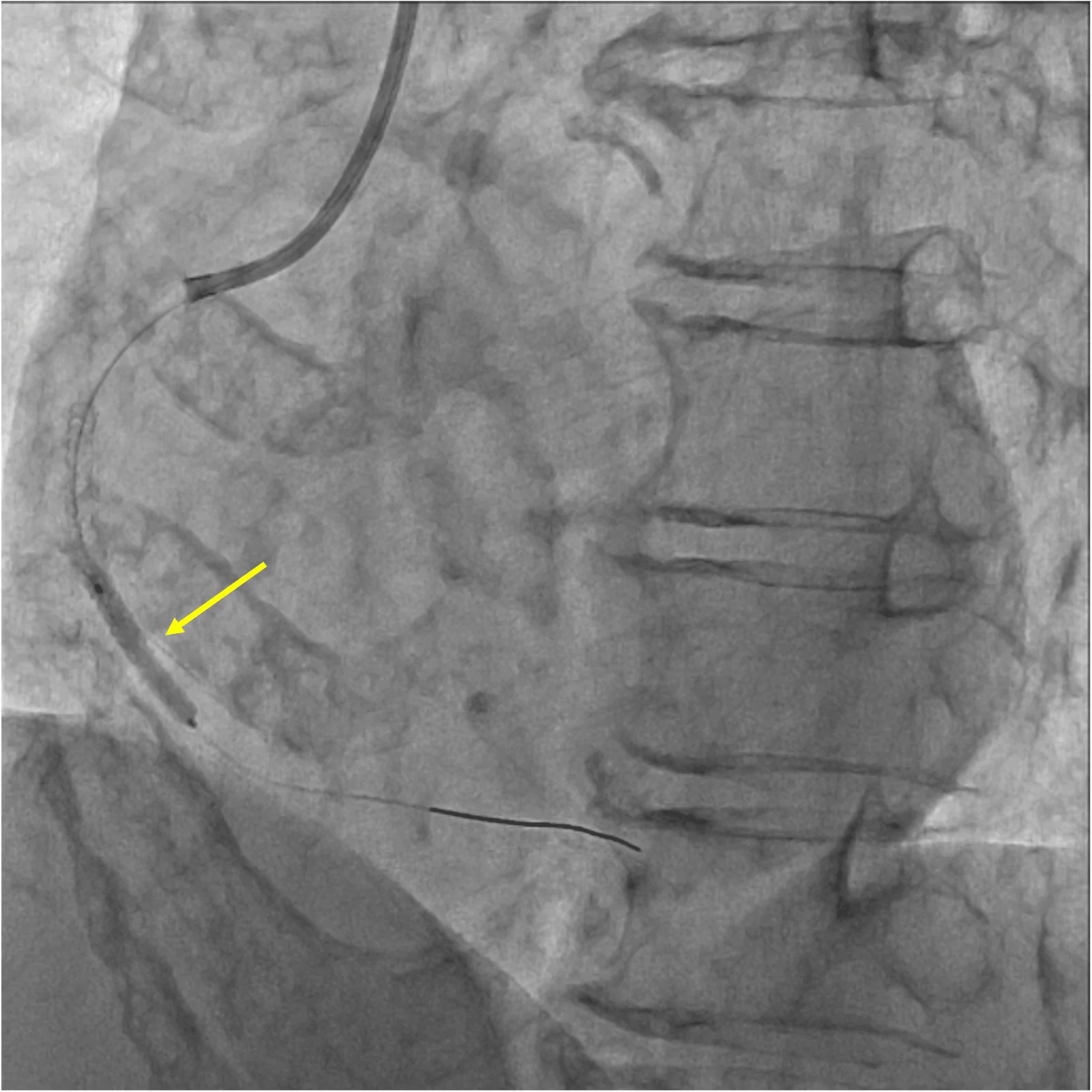
Additional stent implantation at the distal site. After successful guidewire retrieval, a 2.75 × 18-mm drug-eluting stent was additionally implanted at the distal portion of the previously implanted stent (yellow arrow).

Furthermore, coronary dissection developed at the proximal portion of the stent after the bailout procedure. Based on the procedural findings, deep seating of the guiding catheter during repeated device manipulation was considered the most likely cause of the dissection, although the precise mechanism could not be definitively established. A 2.75 × 23-mm DES was therefore implanted at the proximal site (Figure 13). Final IVUS examination after implantation of the additional stents showed no significant stent underexpansion, malapposition, or other major abnormalities. Final coronary angiography demonstrated preserved distal coronary flow (Figure 14).

**Figure 13:**
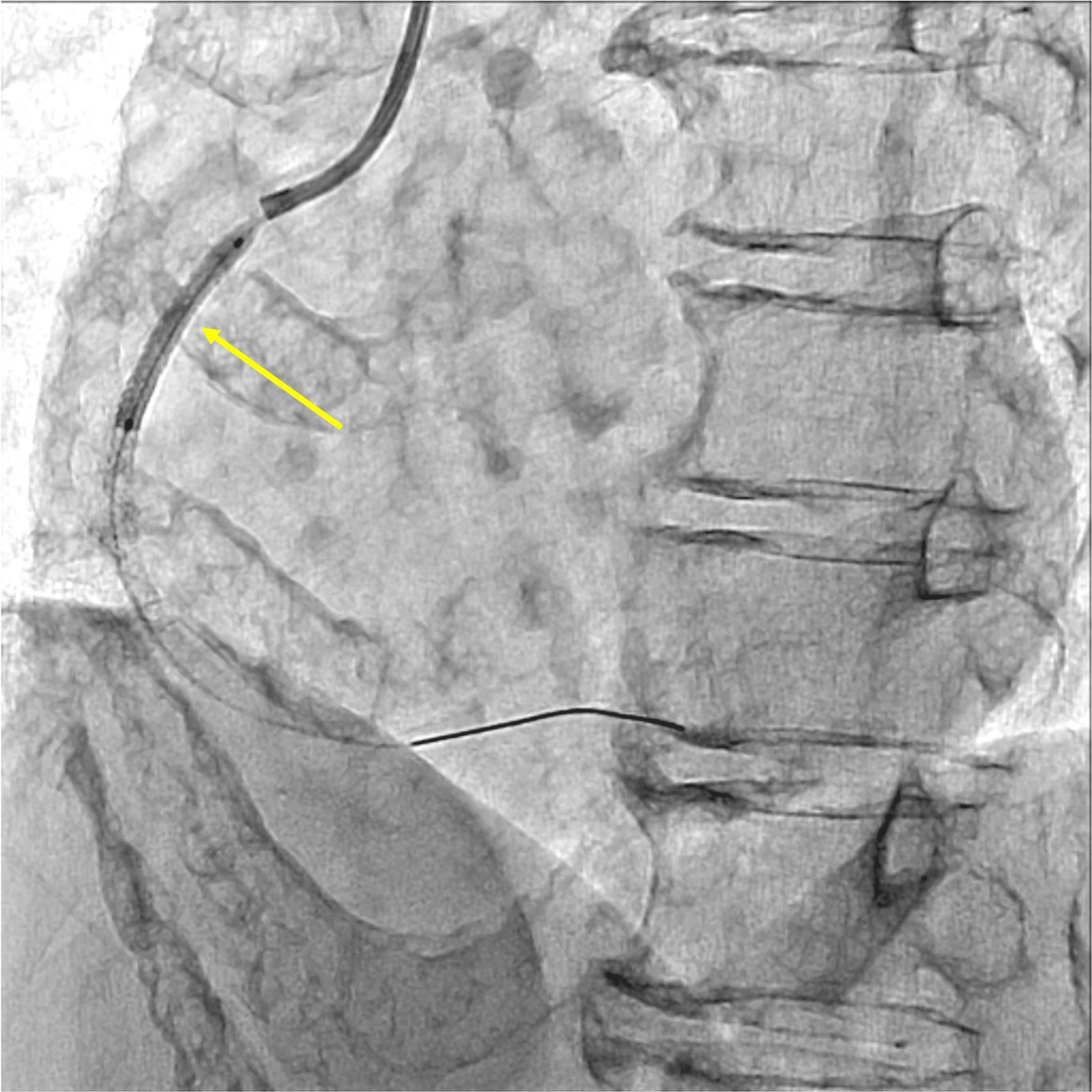
Additional stent implantation at the proximal site. A 2.75 × 23-mm drug-eluting stent was additionally implanted at the proximal portion of the previously implanted stent to treat the coronary dissection (yellow arrow).

**Figure 14:**
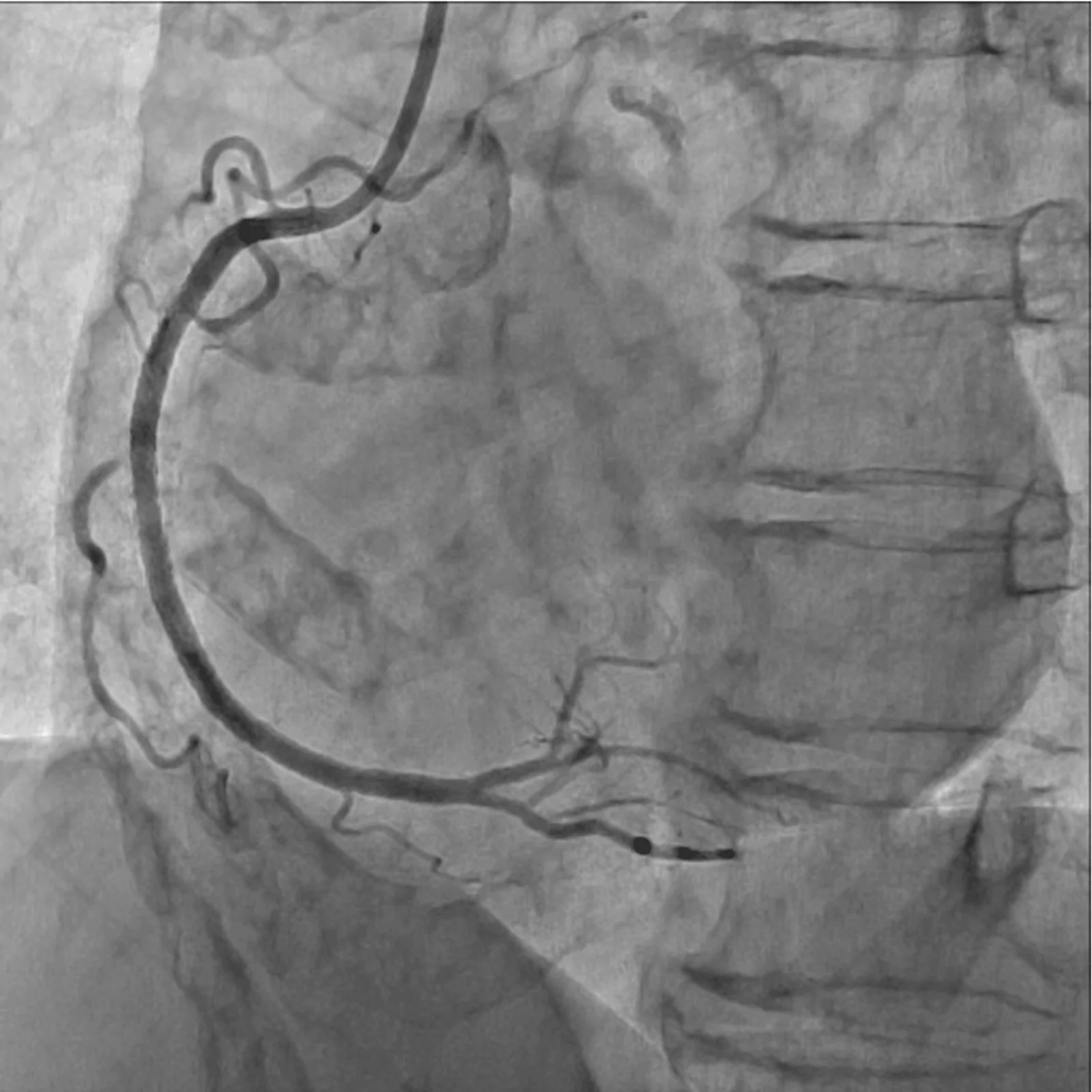
Final coronary angiography after additional stent implantation showed a satisfactory angiographic result with preserved distal coronary flow.

The peak creatine kinase and creatine kinase-MB (CK-MB) levels after the procedure were 784 U/L and 103 ng/mL, respectively. No further in-hospital complications occurred, and the patient was discharged home approximately 10 days after PCI. She was subsequently followed at three-month intervals for one year, during which no major adverse cardiovascular events were observed.

## Discussion

Guidewire entrapment is a rare but potentially serious complication of PCI [1,4]. Although various percutaneous retrieval techniques have been described, including microcatheters, balloon catheters, snares, multiple-guidewire techniques, and those using guide-extension catheters, the optimal strategy depends on the mechanism and location of entrapment [1,2]. Excessive traction on an entrapped guidewire should be avoided because it may result in guidewire fracture, coronary artery injury, or further deformation of an implanted stent [1,2]. In the present case, the guidewire became firmly entrapped within a deployed and deformed coronary stent, making forceful withdrawal particularly undesirable.

Current expert consensus recommends a stepwise percutaneous approach to guidewire entrapment [2]. Advancement of a microcatheter as close as possible to the entrapment site is one of the initial strategies, and balloon inflation around the entrapped segment may also facilitate guidewire release. Rotational microcatheters, including the Tornus catheter, may be useful when conventional microcatheter- or balloon-based techniques are unsuccessful [1,2]. In the present case, we initially attempted guidewire retrieval with a microcatheter, followed by inflation of a 0.75-mm balloon catheter at the site of entrapment. Neither maneuver released the guidewire.

The use of a Tornus catheter for guidewire entrapment has previously been reported. Cho et al. described successful retrieval of an entrapped guidewire using a Tornus catheter in a severely calcified coronary lesion [3]. More recently, Ding et al. reported guidewire entrapment beneath a deployed coronary stent, for which conventional retrieval techniques were unsuccessful and a multiwire technique was ultimately used for bailout [5]. These reports illustrate that the mechanical conditions of guidewire entrapment may differ substantially according to the underlying anatomy. In a severely calcified lesion, resistance to guidewire retrieval may be predominantly determined by the rigid surrounding plaque, whereas entrapment beneath otherwise intact stent struts involves interaction with a relatively preserved metallic scaffold [3,5]. In contrast, entrapment within a deformed stent creates an irregular and mechanically unstable interface in which traction or rotational force may be transmitted not only to the guidewire but also directly to distorted stent struts. The implanted stent in the present case was a XIENCE Skypoint, which has an L-605 cobalt-chromium platform with a strut thickness of 0.0032 inch (approximately 81 μm). However, the mechanical behavior of a deformed stent cannot be inferred from alloy composition or strut thickness alone and may also depend on cell geometry and the specific three-dimensional deformation pattern at the site of entrapment. Because these latter structural parameters could not be fully characterized in the present case, their precise contribution to guidewire disengagement or further stent deformation remains uncertain.

The precise mechanism by which the Tornus catheter facilitated guidewire release remains uncertain. We speculate that rotational advancement of the Tornus catheter generated forces in different directions at the site of entrapment (Figure 15).

**Figure 15:**
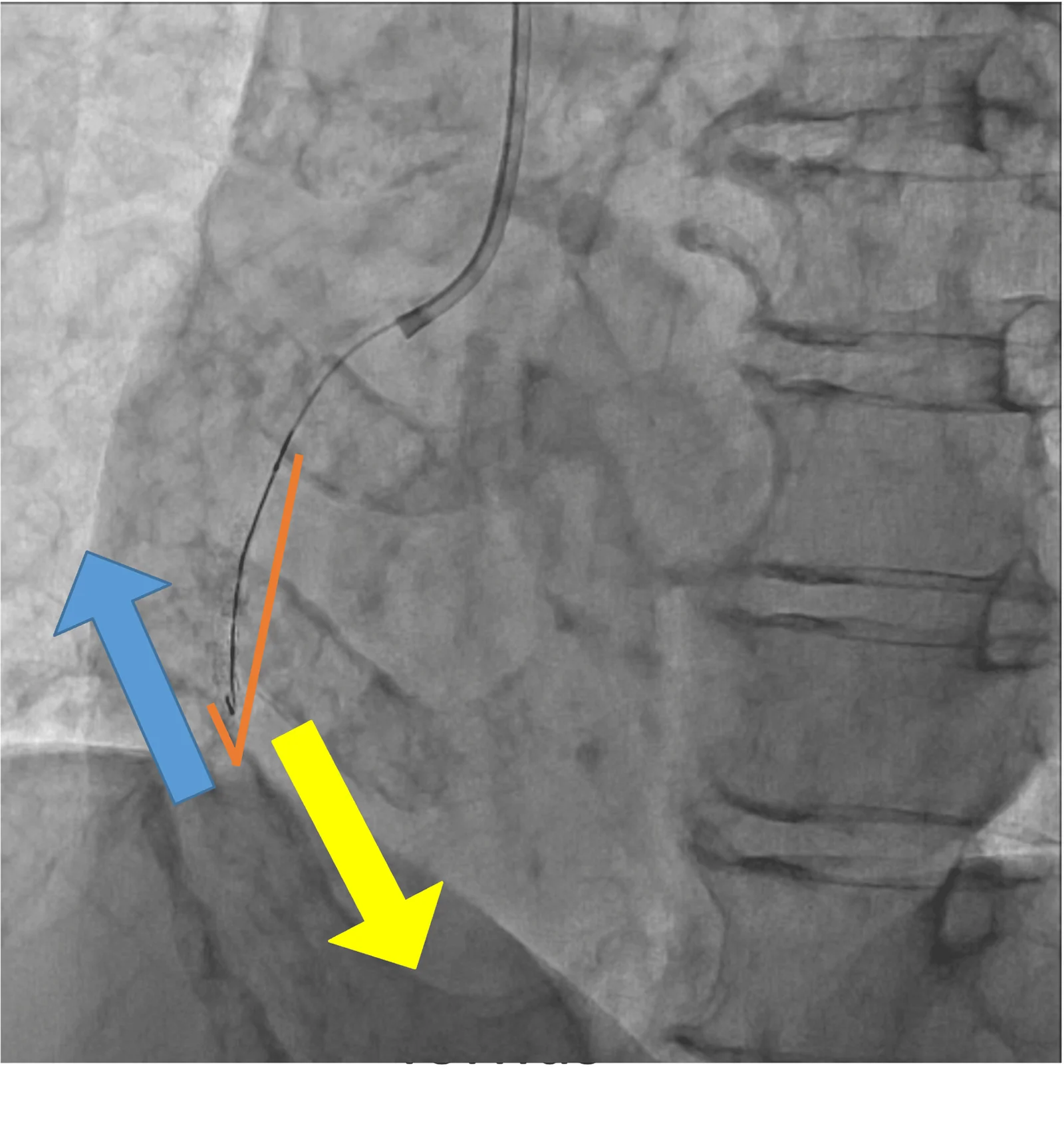
Proposed mechanism of guidewire disengagement using the Tornus penetration catheter. Schematic representation of the proposed forces generated during rotational advancement of the Tornus catheter. Advancement of the catheter generated force along the catheter shaft (yellow arrow), whereas the catheter tip may have exerted force in a different direction at the site of guidewire entrapment because of catheter and coronary curvature (blue arrow). The orange arrow indicates the Tornus catheter. The combination of these forces and rotational torque may have facilitated disengagement of the guidewire from the deformed stent.

While advancement of the catheter exerted force along the catheter shaft, the catheter tip may have exerted force in a different direction against the entrapped segment because of the curvature of the coronary artery and catheter. The combination of these forces, together with rotational torque, may have altered the relationship between the guidewire and the deformed stent struts and thereby facilitated disengagement of the guidewire. Guidewire-specific properties, including its material and coating characteristics, may also influence the mechanical response to traction and rotational manipulation; however, their contribution to guidewire shearing or disengagement could not be determined in this case. Overall, this proposed mechanism remains speculative and cannot be confirmed from a single case.

Importantly, the same mechanical characteristics that may facilitate guidewire disengagement may also pose procedural hazards. Rotational advancement of a relatively rigid catheter within a deformed stent may transmit rotational and longitudinal forces to the compromised stent architecture, while friction among the catheter, guidewire, and stent struts may generate additional shear stress. Such forces could potentially exacerbate stent deformation, contribute to strut fracture, or cause coronary injury. Device entrapment during complex PCI may require multiple retrieval maneuvers and can be associated with procedural complications [6]. In the present case, distal stent fracture was identified after successful guidewire disengagement. Based on the procedural sequence, we considered that structural disruption of the distal stent likely occurred when the guidewire and IVUS catheter became entrapped within the already deformed stent, rather than being caused directly by subsequent Tornus manipulation. However, because the exact timing of the fracture could not be definitively established, a contributory effect of rotational torque, lateral tip force, or associated shear stress during Tornus advancement cannot be completely excluded. The proximal coronary dissection was considered most likely to have resulted from deep seating of the guiding catheter during repeated device manipulation. These observations highlight the biomechanical trade-off inherent in rotational bailout techniques: although multidirectional forces may facilitate guidewire disengagement, they may also impose additional mechanical stress on already compromised stent architecture and the coronary artery. Therefore, a rotational penetration catheter should not be regarded as an automatic next step after failure of conventional retrieval techniques; its use should be considered selectively, with careful advancement under fluoroscopic guidance and avoidance of excessive rotational or longitudinal force.

This case illustrates the feasibility, as well as the potential hazards, of rotational catheter use for guidewire entrapment within a deformed coronary stent. Although rotational advancement of a Tornus penetration catheter facilitated guidewire disengagement after conventional microcatheter- and balloon-based approaches had failed, this single case does not establish the safety or general applicability of this technique. No validated threshold for maximum rotational torque, anatomical contraindication, or quantitative procedural limit can be derived from this case. Continued rotational advancement should be avoided if resistance increases, the device fails to advance despite rotation, further stent deformation is observed, or angiographic or intravascular imaging suggests coronary injury. Under such circumstances, further percutaneous manipulation should be reassessed, and alternative retrieval strategies or surgical consultation should be considered according to the clinical situation. The decision to continue, discontinue, or escalate treatment should therefore be individualized according to the anatomical setting, device behavior, evidence of vascular or stent injury, and intraprocedural imaging findings.

## Conclusions

Guidewire entrapment within a deployed and deformed coronary stent is a rare but challenging complication of PCI. In this case, conventional microcatheter- and balloon-based approaches were unsuccessful, whereas careful rotational advancement of a Tornus penetration catheter to the site of entrapment successfully released the guidewire. This case suggests that rotational advancement of a penetration catheter may facilitate guidewire disengagement when conventional retrieval approaches fail; however, its safety and general applicability remain uncertain, particularly given the distal stent fracture and proximal coronary dissection observed during the procedural course. Increasing resistance, failure of device advancement, further stent deformation, or evidence of coronary injury should prompt discontinuation and reassessment of further rotational manipulation, with consideration of alternative retrieval strategies or surgical consultation when appropriate.

## References

[1] Al-Moghairi AM, Al-Amri HS (2013). Management of retained intervention guide-wire: a literature review. Curr Cardiol Rev.

[2] Ogawa T, Sakakura K, Sumitsuji S (2025). Clinical expert consensus document on bailout algorithms for complications in percutaneous coronary intervention from the Japanese Association of Cardiovascular Intervention and Therapeutics. Cardiovasc Interv Ther.

[3] Cho YH, Park S, Kim JS, Lee SY, Choi D, Jang Y, Cho SY (2007). Rescuing an entrapped guidewire using a Tornus catheter. Circ J.

[4] Lotan C, Hasin Y, Stone D, Meyers S, Applebaum A, Gotsman MS (1987). Guide wire entrapment during PTCA: a potentially dangerous complication. Cathet Cardiovasc Diagn.

[5] Ding Z, Chang S, Wu M, Qin Q, Xu R, Ma J, Ge J (2026). A bailout multiwire technique for retrieval of a deployed coronary stent entrapping a guidewire. JACC Case Rep.

[6] Gasparini GL, Sanz-Sanchez J, Regazzoli D (2021). Device entrapment during percutaneous coronary intervention of chronic total occlusions: incidence and management strategies. EuroIntervention.

